# Effects of chitosan‐based coatings on storage quality of Chinese shrimp

**DOI:** 10.1002/fsn3.1275

**Published:** 2019-11-19

**Authors:** Zhen Zhang, Guanghui Xia, Qiang Yang, Xinwen Fan, Shuxia Lyu

**Affiliations:** ^1^ College of Food Science Shenyang Agricultural University Shenyang China; ^2^ College of Bioscience and Biotechnology Shenyang Agricultural University Shenyang China

**Keywords:** Chinese shrimp, Chitosan‐based coatings, Refrigeration, shelf‐life, storage quality

## Abstract

We investigated the effects of chitosan‐based coatings on the preservation quality of refrigerated Chinese shrimp for 12 days. Samples of Chinese shrimp were subjected to three different coating treatments, namely chitosan (CH), chitosan and ε‐polylysine (CH + ε‐PL), chitosan combined with ε‐polylysine and carrageenan (CH + ε‐PL + CA), and compared with a control. The bacteriological characteristics [total viable count (TVC)], chemical indexes including pH, thiobarbituric acid (TBA) value, K‐value, and total volatile basic nitrogen (TVB‐N), texture (hardness, chewiness, and elasticity), and sensory changes were assessed. The increases in TVC, pH, TBA, K‐value, and TVB‐N were observed to be delayed by preservation treatments, and the textural and sensory characteristics indicated that the treated shrimp were preserved more effectively than the control. Treatment with chitosan combined with ε‐polylysine and carrageenan was the most effective preservation method than treatment with chitosan alone or chitosan and ε‐polylysine; the shelf life was also prolonged. Therefore, treatment with chitosan combined with ε‐polylysine and carrageenan is proposed as a potential method for shelf life extension of Chinese shrimp for refrigerated storage.

## Graphical abstract

The growth of microorganism, decomposition of protein, and oxidation of lipid could be effectively inhibited by chitosan‐based coating treatment. Treatment with chitosan combined with ε‐polylysine and carrageenan is proposed as a potential method for shelf life extension of Chinese shrimp for refrigerated storage.

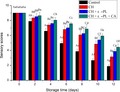

## 1 INTRODUCTION

Chinese shrimp (*Fenneropenaeus chinensis*) is one of the three famous varieties of shrimp globally and is primarily cultured in the Bohai Sea and Yellow Sea in China; it is also distributed in the western sea region of North Korea. Shrimp is an important aquatic export product in China, and the production of Chinese shrimp was reportedly 37,500 tons in 2017 (Anonymous, 2018). Because of its tender and delicious meat, rich polyunsaturated fatty acids, high protein, low fat, and variety of vitamins and essential trace element content (Lu, 2009), Chinese shrimp is considered to be an ideal food product with important implications for human health and is welcomed by the international market. However, Chinese shrimp are easily perishable, and the shelf life is very short owing to their high protein and water contents, autolysis, and microbial metabolism (Li, Yang, & Li, 2017). The deterioration of Chinese shrimp causes production of black spots (melanosis), accumulation of putrid compounds, and deterioration in textural quality, as well as the loss of essential fatty acids and proteins. The original sensory characteristics, flavor, and nutritional value of the shrimp are also lost. As a consequence, their market and economic value are greatly reduced. Therefore, preventing shrimp spoilage and maintaining its original flavor and sensory quality are of great significance. A variety of preservation techniques, such as packaging, freezing, and chemical preservation, have been used to preserve shrimp quality (Zhang, Ma, Deng, Xie, & Qiu, 2015). Among them, chemical preservation is one of the universal and effective methods to maintain quality and prolong the shelf life of shrimp during storage. However, because of consumers' dislike of synthetic chemicals, recent studies have shown a growing interest in extending the shelf lives of seafood using natural extracts with antimicrobial and antioxidant properties rather than synthetic additives (Dehghani, Hosseini, & Regenstein, 2018).

Edible coatings are garnering more attention owing to their safety, efficacy, and cost and have proven to be an effective way to maintain the quality of seafood (Yu et al., 2018). Coating materials used for preservation of seafood are limited. Besides safety, they must be able to serve as carriers for seafood preservatives such as antioxidants and antimicrobial agents. Potential materials include hydrocolloids, lipids, and composites; chitosan and carrageenan are the two most commonly used polysaccharides in hydrocolloids (Dehghani et al., 2018). Chitosan (CH), a widely present polysaccharide, is a derivative of chitin by deacetylation. It can be absorbed by the body and is the only high‐molecular‐weight alkaline polysaccharide that is widely abundant in nature (Aider, 2010). Because of its excellent characteristics, such as film‐forming and antibacterial properties (Günlü & Koyun, 2013), capability to prevent microbial degradation, and biocompatibility, chitosan has been widely used as an edible coating material to maintain the quality of seafood by inhibiting the growth of microorganisms, decomposition of proteins, and oxidation of lipids during storage (Günlü & Koyun, 2013; Soares, Fernandes, & Vicente, 2016). ε‐polylysine (ε‐PL), a cationic homopolymer of 25–35 L‐lysine units linked by an amide linkage between the ε‐amino and carboxyl groups, is stable in both acidic and alkaline environments (Lin, Liao, Surendhiran, & Cui, 2018). This compound has been used as an antimicrobial preservative for food (Li et al., 2014) because of the broad‐spectrum antimicrobial properties, nontoxicity, and biocompatibility (Wu et al., 2019). In addition, ε‐polylysine is a nutrient‐type bacteriostatic agent that can be decomposed into lysine—one of the eight essential amino acids (Yoshida & Nagasawa, 2003). In United States, Japan, and Korea, ε‐polylysine has been approved as a food preservative (Jia et al., 2019). Carrageenan (CA), another natural polysaccharide extracted from marine red algae, has been widely used as a coating material (Dehghani et al., 2018) or base material for edible films owing to its good film‐forming, renewable, and biocompatible properties (Roy & Rhim, 2019). Carrageenan has the basic properties of soluble dietary fibers; it can form soluble complexes with fibrin after degradation in vivo, and the protein stability, emulsifying properties, gel strength, and antioxidant properties could be enhanced (Tavassoli‐Kafrani, Shekarchizadeh, & Masoudpour‐Behabadi, 2016). Studies have shown that carrageenan, as a novel natural coating material, has been used for the preservation of ham (Carocho et al., 2019) and maintain the color of lychee (Plotto, Narciso, Baldwin, & Rattanapanone, 2006).

In recent years, chitosan combined with ε‐polylysine has been used in the prevention of white blushing of carrots (Song et al., 2017), citrus fruits (Li, Ye, Hou, & Zhang, 2018), and chicken (Lin et al., 2018). Coating with chitosan combined with ε‐polylysine and rosmarinic acid effectively maintains the quality of half‐smooth tongue sole fillets (Li, Liu, Shen, Mei, & Xie, 2019). Chitosan combined with carrageenan and gelatin has also been used to encapsulate peppermint oil (Irma, Qurrota, Hanina, & Marsasi, 2019)). Moreover, it has been shown that ε‐polylysine has a good interaction with carrageenan gels (Li et al., 2019). However, there are very few reports, if any, on the quality preservation of seafood products by chitosan combined with ε‐polylysine and carrageenan. In this study, referring to the reports on the usage amount of chitosan by Yu et al. (2018), ε‐polylysine by Jia et al. (2019), carrageenan by Bico, Rapaso, Morais, and Morais (2009), and pre‐experiments on their preservation effects on Chinese shrimp, 1.8% chitosan, 1.8% chitosan with 0.13% ε‐polylysine, and 1.8% chitosan with 0.13% ε‐polylysine and 0.19% carrageenan were prepared as the chitosan‐based coatings; their efficacies for quality maintenance of Chinese shrimp during refrigerated storage were explored via microbiological, chemical, textural, and sensory indices.

## 2 MATERIALS AND METHODS

### 2.1 Samples and chemicals

Chinese shrimp (*Fenneropenaeus chinensis*), which had a size corresponding to 50–55 shrimp/kg, were obtained from Jinzhou aquatic market, Liaoning, China, and transferred to the Food Processing Laboratory of Jinzhou Medical University within 30 min and kept alive. After killing, washing, and draining, the samples were randomly divided into four treatment groups. The chemicals used include chitosan (85% deacetylation degree, food grade; Zhejiang Aoxing Biotechnology Co., Ltd., Zhejiang, China), ε‐polylysine (Shandong Xin Ding Biotechnology Co., Ltd., Shandong, China), carrageenan (Henan Hongxin Food Co., Ltd., Henan, China), thiobarbituric acid (Beijing Sola Po Technology Co., Ltd., Beijing, China), 1,1,3,3‐tetraethoxypropane (Kelvin Chemical Technology (Beijing) Co., Ltd., Beijing, China), hydrochloric acid, glacial acetic acid, EDTA, magnesium oxide, and boric acid (Tianjin Wind Boat Chemical Reagents Technology Co., Ltd., Tianjin, China).

To prepare the coating solutions, chitosan (18 g) was poured into a flask containing 1% (v/v) acetic acid (700 ml). It was stirred until completely dissolved, and the volume was then filled to 1,000 ml. Briefly, to obtain the CH + ε‐PL coating solution containing 1.8% chitosan and 0.13% ε‐polylysine, 1.3 g of ε‐polylysine was mixed with the prepared CH coating solution. CH + ε‐PL + CA coating solution was obtained by adding 0.19 g carrageenan into the prepared CH + ε‐PL solution.

The Chinese shrimp samples were dip‐treated in a solution containing 1.8% chitosan (CH group), 1.8% chitosan and 0.13% of ε‐polylysine (CH + ε‐PL group), 1.8% chitosan with 0.13% ε‐polylysine and 0.19% carrageenan (CH + ε‐PL + CA group), and distilled water (control group) for 1 hr. After draining at 4℃ for 30 min on sterile metal nets, they were packed in air‐proof polyethylene pouches and stored at 4℃. The bacteriological, physicochemical, and sensory characteristics were evaluated for all samples in 2‐day intervals.

### 2.2 Bacteriological analyses

Each sample (10 g) and 0.85% NaCl solution (90 ml) was transferred to a stomacher and homogenized for 1 min. Other serial dilutions were prepared from this dilution; the total viable counts (TVC) were determined by plate count agar (PCA, Base Bio‐Tech, Hangzhou, China) after incubation at 30°C for 48 hr.

### 2.3 Chemical analyses

#### 2.3.1 pH

The pH was measured according to the Chinese National Standard ((GB/T 5009.45‐2003)). Briefly, 5 g of chopped shrimp was homogenized in 50 ml of deionized water for 30 min. After filtering, the pH of the supernatant was recorded with a digital PHS‐3C pH meter (Shanghai INESA Scientific Instrument Co., Ltd., China).

#### 2.3.2 Thiobarbituric acid reactive substances (TBARS)

TBARS was measured as described by Kirk and Sawyer (1991). A small amount (5 g) of the sample was mixed with 0.2g/ml trichloroacetic acid (25 ml) and distilled water (20 ml) using a 100‐mL conical flask, shaken for 30 min, filtered with double filter paper, and the grease was removed. After repeated filtering, the filtrate (5 ml) and TBA solution (5 ml) were thoroughly mixed in a 25‐ml colorimetric tube and sealed with a stopper. After heating in a 90°C water bath for 40 min, the resulting mixture was cooled to room temperature with flowing water and centrifuged for 5 min. The absorbance was measured at 532 nm. The TBARS value was expressed as mg malondialdehyde (MDA) • kg^−1^ sample.

#### 2.3.3 K‐value

The K‐value was determined using the China Fisheries Industry Standard ((SC/T 3048‐2014)). Extraction of ATP‐related compounds from the Chinese shrimp was performed as follows: The homogenized sample (2 g) and 10% perchloric acid solution (20 ml) were mixed, vortexed for 1 min, and centrifuged at 8,000 r/min for 10 min at 4°C. After collecting the supernatant, the analyte was extracted from the precipitate twice according to the above process parameters. The pH of the combined supernatant was adjusted within the range of 6.0–6.4 using 1.0 mol/L NaOH. The pH‐adjusted solution was transferred to a precooled volumetric flask, made up to 50 ml, centrifuged at 8,000 r/min at 4°C for 10 min, and filtered with a 0.22 μm microporous membrane.

The following HPLC conditions were used for ATP correlation analyses: C_18_ column (250 mm × 4.6 mm, 5 μm) mixed with 0.02 M potassium dihydrogen phosphate and 0.02 M dipotassium phosphate 1:1 liquid as the mobile phase. The flow rate of the sample (10 µl) was maintained 1 ml/min, and the peak was detected at 254 nm.

The K‐value was computed as the ratio of the total amount of inosine and hypoxanthine degraded by inosine triphosphate to the sum of adenosine triphosphate‐related compounds as follows:

$$K-\text{value}\%=\frac{\text{MHxR}\;+\;\text{MHx}}{\text{MATP}+\text{MADP + MAMP + MIMP + MHxR + MHx}}\times 100$$

HxR: hypoxanthine adenosine, Hx: hypoxanthine, ATP: adenosine triphosphate, ADP: adenosine 5’‐diphosphate, AMP: adenosine 5’‐monophosphate, and IMP: inosine 5’‐monophosphate.

#### 2.3.4 TVB‐N

TVB‐N was measured by referring to the Chinese National Standard (GB 5009.228‐2016). Briefly, 10 g of crushed shrimp was mixed with 100 ml of distilled water in a conical flask, homogenized, and filtered. Next, 5 ml of the filtrate and 5 ml MgO suspension were injected into the reaction chamber. Boric acid solution (10 ml) and a mixture of methyl red and methylene blue (v:v = 2:1) were added and distilled. Afterward, the boric acid solution was titrated with 0.1 mol/L HCl solution. The TVB‐N value, which was expressed in mg nitrogen (mg/100 g) for 100 g^−1^ shrimp sample, was determined based on the consumption of HCl solution.

### 2.4 Texture properties analysis (TPA)

TPAs were carried out according to Sigurgisladottir et al. (1999). TPA was evaluated by hardness, elasticity, and chewiness using a Texture Analyzer (Model number TMS‐Pro, Beijing Yingsheng Hengtai Technology Co., Ltd., China). Six samples from three shrimps were used for analysis. The flat bottom cylindrical probe (50 mm diameter) was used. The parameters were set as follows: initial strength, 0.8 N; probe recovery height, 25 mm; test speed, 60 mm/min; and sample compression rate, 30%.

### 2.5 Sensory evaluation

The sensory evaluation of Chinese shrimp was performed by a well‐trained panel of seven evaluators on each sampling day. Each assessor scored the characteristics from 1 to 9 with regard to color, texture, odor, and overall acceptability of the shrimp (9 = most ideal, 1 = lowest quality). The shrimp was considered acceptable until the sensory score dropped to 5.0 (Yao, Chang, Wu, 2015).

### 2.6 Statistical analysis

Data except TPA and the sensory evaluation were expressed as mean ± standard error of the mean (*n* = 3). Analyses were performed using SPSS software (Version 19.0; SPSS, Chicago, IL). *p* < .05 was considered statistically significant.

## 3 RESULTS AND DISCUSSION

### 3.1 Bacteriological analysis

**Figure 1:**
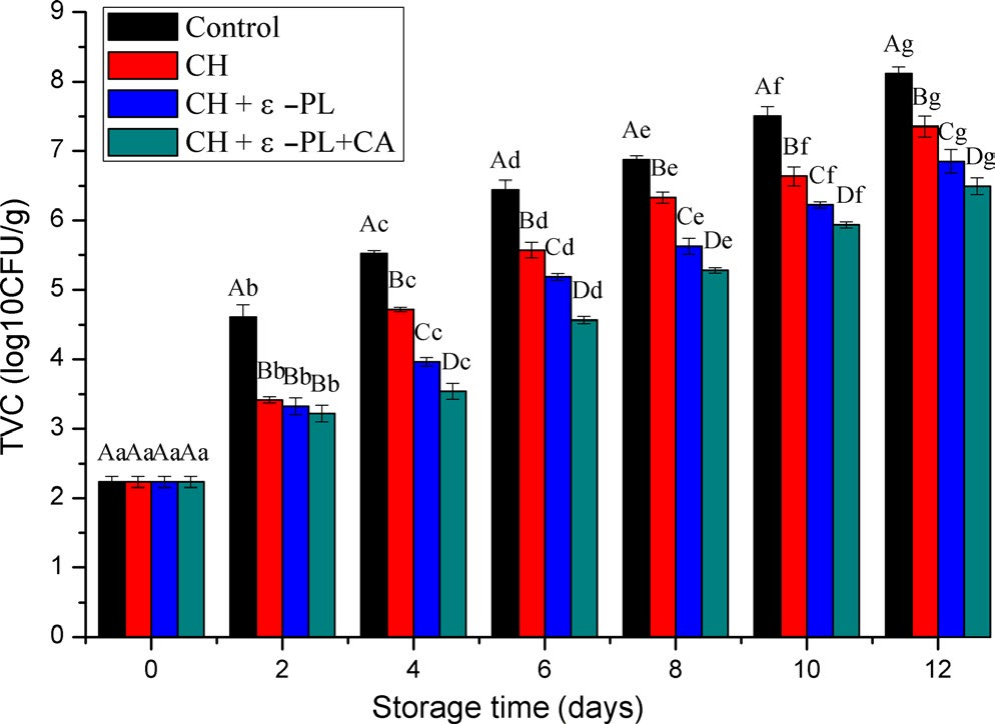
Changes in total viable counts (TVCs) of Chinese shrimp during refrigerated storage. Different lowercase letters on the bars within the same sample denote significant difference (*p* < .05). Different uppercase letters on the bars within the same storage time denote significant difference (*p* < .05)

Microbial metabolites significantly contribute to undesired changes in odor, texture, and appearance of seafood products. As a result, many countries have adopted TVC as a mandatory test indicator for seafood quality (Li, Li, Hu, & Li, 2013). According to previous studies, the sample is considered fresh grade if the TVC is lower than 5.0 log CFU/g, while it is extremely spoiled when the value increases above 6.0 log CFU/g (Al‐Daqal & Bazaraa, 1999). In this study, the observed TVC changes are shown in Figure 1. The TVC of shrimp samples with the same treatment significantly increased during 12‐day storage for all the four groups. The low initial of TVC (2.23 ± 0.08 log CFU/g), indicating that the shrimp samples were fresh. The TVC of control group gradually increased and reached 5.52 ± 0.04 and 6.44 ± 0.14 log CFU/g on the 4th and 6th day, respectively, indicating that the microbial shelf lives of the control samples ranged about 4–6 days. The TVC values of CH group, CH + ε‐PL group, and CH + ε‐PL + CA groups exceeded the 6.0 log CFU/g on the 8th, 10th, and 12th day, respectively. The increase in TVC was delayed in samples subjected to CH treatment (*p* < .05), which was related to the broad antibacterial properties of chitosan (Ngo et al., 2015), was consistent with the results previously reported by Xu et al. (2014). The current findings additionally indicated that the antimicrobial effects of chitosan were significantly enhanced by the incorporation of 0.13% ε‐polylysine and 0.19% carrageenan into the chitosan coating. ε‐polylysine is a kind of microbial food preservative that exhibits strong antibacterial properties against both bacteria and yeast as well as mold. Though carrageenan itself does not have antibacterial properties, its good film‐forming property and biocompatibility (Roy et al., 2019) render the composite coating on the surface of the shrimp more effectively capable of inhibiting microbial proliferation; therefore, the lowest TVC was seen in the CH + ε‐PL + CA group. The addition of other natural preservatives, such as citric acid, pomegranate peel extract (Yuan, Lv, Tang, Zhang, & Sun, 2016), maqui berry (Genskowsky et al., 2015), and tea polyphenols (Li, Hu, Li, Zhang, Zhu, & Li, 2012), can also enhance chitosan's antibacterial abilities. In the present study, chitosan incorporated with ε‐polylysine and carrageenan presented stronger antibacterial effects and showed improved preservation performance on Chinese shrimp compared to chitosan alone or chitosan combined with ε‐polylysine.

### 3.2 Chemical analyses

#### 3.2.1 pH

**Figure 2:**
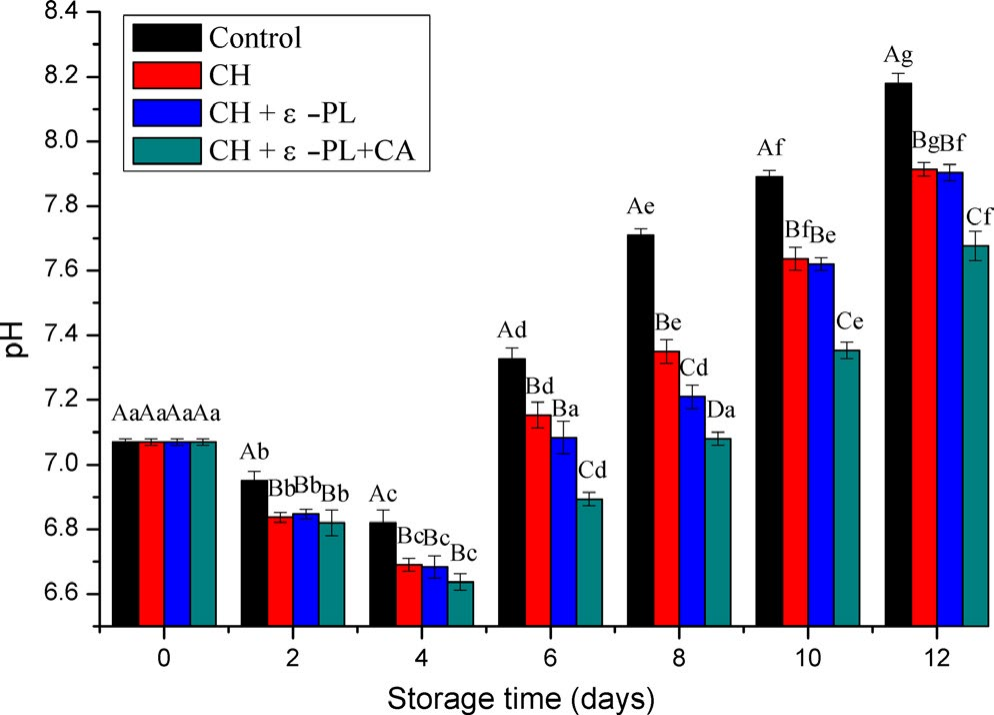
Changes in pH values of Chinese shrimp during refrigerated storage. Different lowercase letters on the bars within the same sample denote significant difference (*p* < .05). Different uppercase letters on the bars within the same storage time denote significant difference (*p* < .05)

Changes in pH values, which are closely related to seafood freshness, have been used as a reliable indicator of shrimp freshness. The recorded changes in the pH throughout storage are given in Figure 2. At day 0, pH value was 7.07 ± 0.01, which agree with 7.04 reported by Mu, Chen, Fang, Mao, and Gao in 2012, but lower than the result from Li et al., who reported the initial pH value was 7.36 in 2017. The differences could be attributed to the season, species, type of muscle, and diet, as well as the level of activity. With prolonged storage, the pH of all samples in the same group decreased significantly (*p* < .05) and then increased gradually, which was in agreement with that reported by Mu, Chen, Fang, Mao, and Gao (2012). The decrease in pH in the early stage is caused by acidic components, such as lactic acid, which is produced by the glycolysis reaction, and phosphoric acid, which is produced by the degradation of ATP and related products (Qiu, Chen, Liu, & Yang, 2014). The pH increase in later stage could be attributed to the presence of alkaline products, including ammonia, trimethylamine, indole, and histamine, which are formed by the decomposition of the proteins, amino acids, and other nutrients by various enzymes and shrimp spoilage bacteria (Hui et al., 2016). According to Mehmet, Faruk, and Hami (2009), the pH of shrimp below 7.7 indicates good quality. The pH in the control group reached 7.71 ± 0.02 at the end of shelf life on day 8. The pH of the treatment groups was significantly lower than those of the controls (*p* < .05) during storage, indicating that treatment with chitosan or composite coating is effective for maintaining quality of Chinese shrimp and extending their shelf lives. These desired properties could be attributed to the antibacterial characteristics of chitosan and ε‐polylysine. The CH and CH + ε‐PL groups showed no significant differences (*p* > .05) in pH throughout the refrigerated period. The pH of samples in the CH + ε‐PL + CA group reached 7.68 ± 0.05 on day 12. Obviously, the pH was consistently lower than those of samples in the CH and CH + ε‐PL groups (*p* < .05). The above results are attributed to the enhanced inhibitory effects of the composite coating on microbial growth and enzyme activity, caused by the addition of carrageenan, which lead to the consequent delay in decomposition of protein or other nutrients. Compared to samples treated with chitosan or chitosan and ε‐polylysine, Chinese shrimp samples treated with a mixture of chitosan, ε‐polylysine and carrageenan coating were more effectively preserved.

#### 3.2.2 TBA

**Figure 3:**
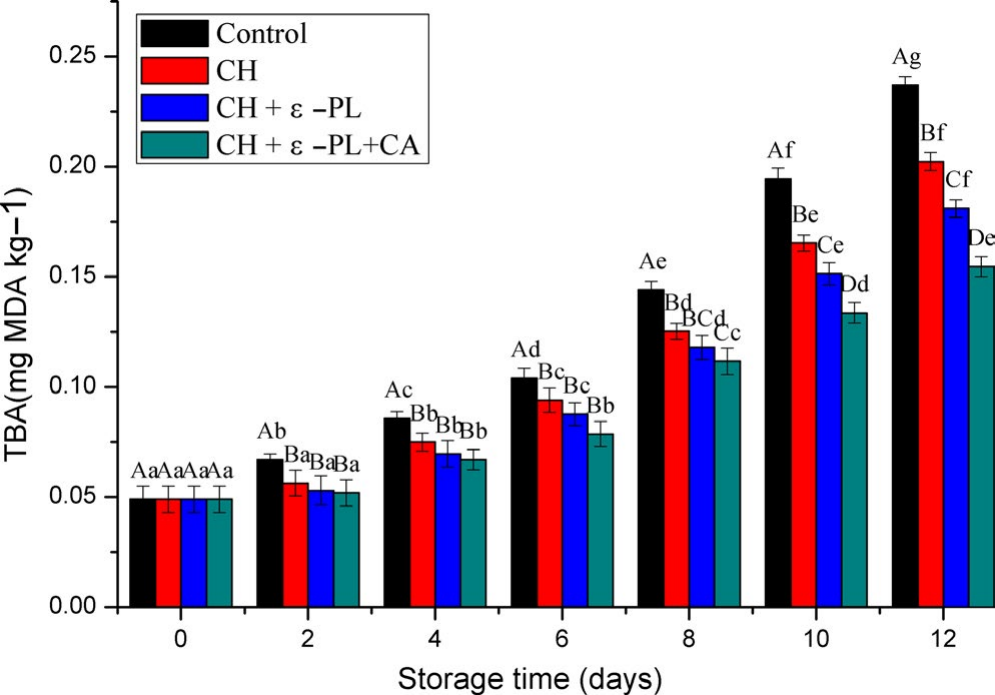
Changes in TBA values of Chinese shrimp during refrigerated storage. Different lowercase letters on the bars within the same sample denote significant difference (*p* < .05). Different uppercase letters on the bars within the same storage time denote significant difference (*p* < .05)

Peroxide is an intermediate product that is generated during the rancidity of fat‐containing foods and can be decomposed into acids, ketones, and some oxides. The TBA (thiobarbituric acid) content has been widely used to evaluate the degree of lipid oxidation (Ojagh, Rezaei, Razavi, & Hosseini, 2010) because of strong correlation, sensitivity, and simplicity of the measurement (Li et al., 2013). The TBA values of all samples subjected different treatments are presented in Figure 3. Results showed that the initial TBA value of Chinese shrimp was 0.049 ± 0.006 mg MDA•kg^−1^, similar to that reported by Kirschnik, Viegas, Valenti, and Oliveira (2006). The slight differences in the TBA values could be attributed to factors such as differences in fat composition and preservation temperature. A low TBA value in shrimp is associated with lower fat content. The TBA values of four treatments continuously increased throughout storage; the increase was significant in the control group from the early storage, but significance was observed from the fourth day in the CH and CH + ε‐PL groups, and there was no significant increase from day 0 to the 2nd day, the 4th day to the 6th day in the CH + ε‐PL + CA group. Compared with the control, they were significantly lower (*p* < .05) in CH, CH + ε‐PL, and CH + ε‐PL + CA groups, while there was no significant difference among CH, CH + ε‐PL, and CH + ε‐PL + CA groups until the 10th day. The final TBA value of CH + ε‐PL + CA group was 0.155 ± 0.004 mg MDA•kg^−1^, and it was 35% lower compared to that of the control, consistent with the results obtain by Ojagh et al. (2010), and Fan, Chi, and Zhang (2008). Several research studies have shown that edible coatings, such as chitosan, could protect seafood against oxidation (De Abreu, Losada, Maroto, & Cruz, 2011; Dehghani et al., 2018). Our current results revealed that lipid oxidation of Chinese shrimp was delayed by chitosan and a composite coating, and samples in the CH + ε‐PL + CA group exhibited stronger inhibition than that of chitosan or chitosan and ε‐polylysine because the CH + ε‐PL + CA acts as a more effective barrier to prevent oxygen diffusion to the surface of shrimp than CH or CH + ε‐PL.

#### 3.2.3 K‐value

**Figure 4:**
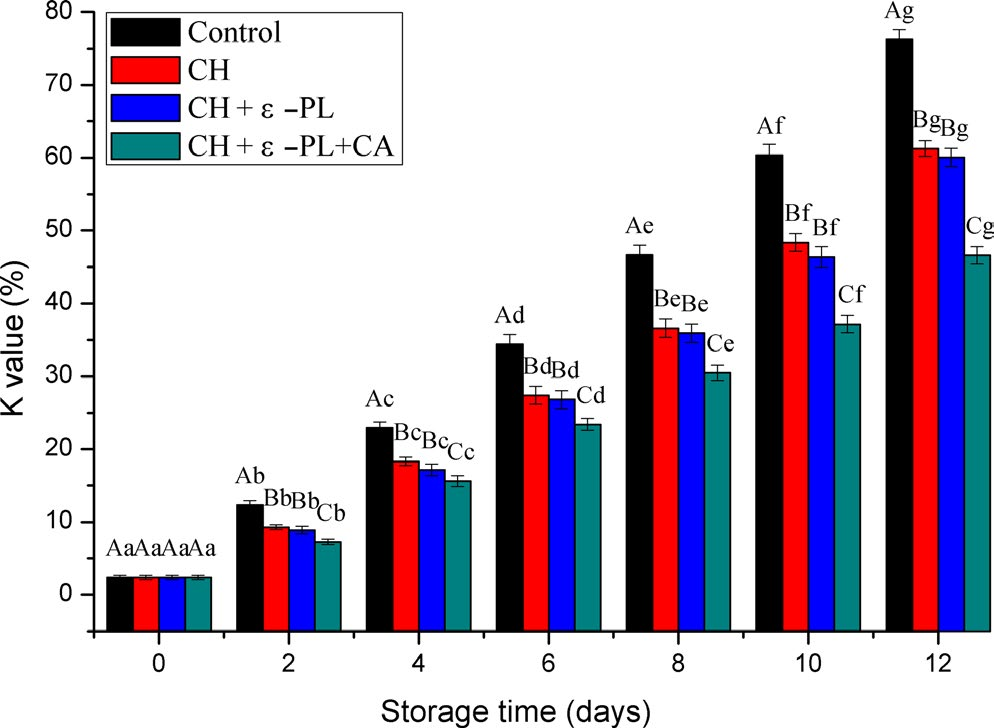
Changes in the K‐values of Chinese shrimp samples during refrigerated storage. Different lowercase letters on the bars within the same sample denote significant difference (*p* < .05). Different uppercase letters on the bars within the same storage time denote significant difference (*p* < .05)

The K‐value is the ratio of the total amount of inosine and hypoxanthine degraded by inosine triphosphate to the sum of adenosine triphosphate‐related compounds, and the index has been frequently used to evaluate freshness in seafood (Ocaño‐Higuera et al., 2011; Pardio, Waliszewski, & Zuñiga, 2011). Previous studies considered seafood with K‐values above 60%, 20%‐50%, and lower than 20% as not fresh, moderately fresh, and very fresh, respectively (Ehira, 1976; Saito, Arai, & Matsuyoshi, 1959). As shown in Figure 4, the K‐value was 2.41 ± 0.32% on day 0, which was slightly higher than 1.80% reported by Ando, Nakamura, Harada, and Yamane (2004), and eventually reached 76.26 ± 1.34% at 12th day. The differences in the K‐value of the aquatic products may due to various factors, such as the species, muscle type, stress during capture, and processing temperature (Alasalvar et al., 2001; Sallam, 2007). A gradual increase in the K‐value was observed in both treated and control groups during the 12‐day storage period, and the increase was significant in the same group; however, the K‐values of samples in the treated groups were significantly lower (*p* < .05) than those of the control samples. Based on the previous classification of K‐values, the samples in the control group cannot be considered fresh on day 10, the samples in the CH and CH + ε‐PL groups were not fresh on day 12, and the samples in the CH + ε‐PL + CA group were moderately fresh at the end of storage, indicating that chitosan, ε‐polylysine, and carrageenan effectively inhibited ATP degradation and extended the shelf lives of the shrimp samples. According to Li et al. (2013), the action of 5‐nucleotidase will cause the inosine monophosphate to decompose, resulting in a lower k‐value. The above result could be attributed to the stronger inhibition on 5‐nucleotidase by chitosan combined with ε‐polylysine and carrageenan than chitosan or chitosan combined with ε‐polylysine.

#### 3.2.4 TVB‐N

**Figure 5:**
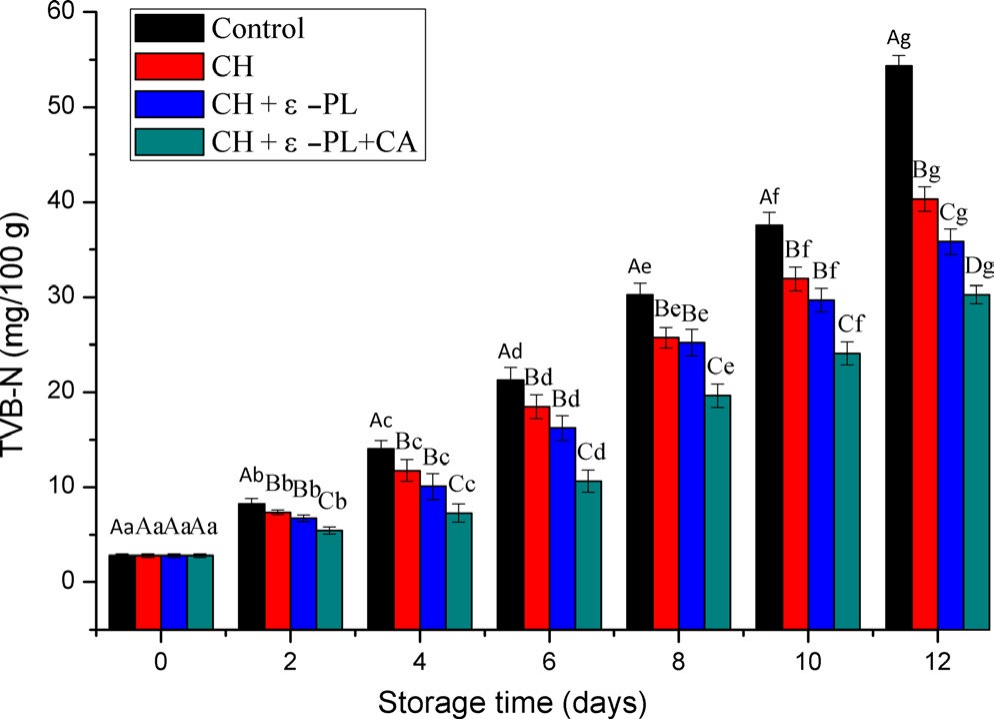
Changes in the TVB‐N values of Chinese shrimp during refrigerated storage. Different lowercase letters on the bars within the same sample denote significant difference (*p* < .05). Different uppercase letters on the bars within the same storage time denote significant difference (*p* < .05)

TVB‐N is used to evaluate the freshness of aquatic products as an important indicator (Hui et al., 2016). According to the hygienic standards for marine products, the acceptable TVB‐N values for marine fish and shrimp are less than 30 mg/100 g ((GB 2733‐2015)). As shown in Figure 5, the TVB‐N values continuously increased from 2.80 ± 0.17 mg/100 g in all samples with prolonged storage, and the increase was significant in the same group; however, it was especially faster (*p* < .05) in the control group than that in the treatment groups. It reached 30.25 ± 1.20 mg/100 g in the control group at 8th day, which was significantly higher (*p* < .05) than those of the treated sample groups (*p* < .05). The TVB‐N values of shrimp samples in the CH+ε‐PL group were slightly lower than those of CH  group, but there was no significant difference (*p* > .05) except the twelfth day, and eventually reached 35.84 ± 1.36 and 40.32 ± 1.28 mg/100 g at 12th day, respectively. The lowest TVB‐N values was showed in the CH + ε‐PL + CA group, which were 24.08 ± 1.19 mg/100 g at 10th day and 30.24 ± 0.95 mg/100 g at 12th day. As a comparison, the TVB‐N values in the control group reached 54.32 ± 1.13 mg/100 g at 12th day. The TVB‐N present in postmortem shrimp is primarily produced via microbial degradation of proteins and other nitrogen‐containing compounds (Li, Li, Hu, Zhang, Li, Zhao, 2012; Song, Liu, Shen, You, & Luo, 2011). In the present study, the reason for the lowest TVB‐N values in the CH + ε‐PL + CA group is that chitosan, ε‐polylysine, and carrageenan could strongly inhibit bacterial growth or reduce the oxidative deamination of nitrogen‐containing compounds by bacteria. The above results were consistent with conclusions reported in previous research, wherein TVB‐N values of half‐smooth tongue sole fillets treated with chitosan ε‐polylysine and rosmarinic acid were found to be lower than those of the control, chitosan or chitosan and rosmarinic acid (Li, Wen, et al., 2019).

### 3.3 Texture properties

#### 3.3.1 Hardness

**Figure 6:**
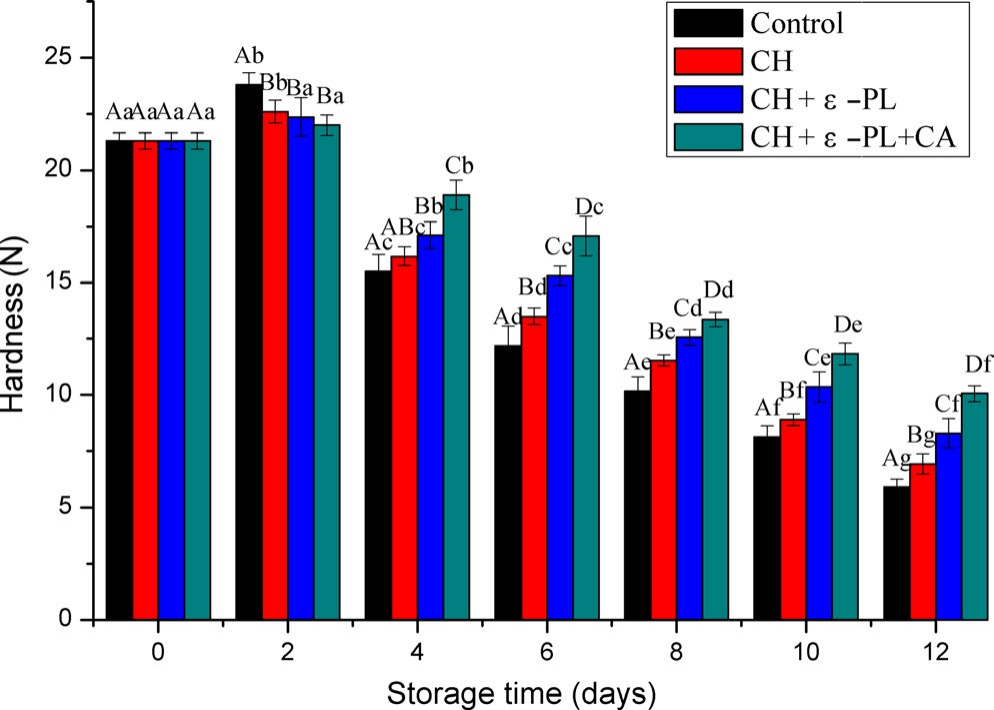
Changes in hardness scores of Chinese shrimp during refrigerated storage. Different lowercase letters on the bars within the same sample denote significant difference (*p* < .05). Different uppercase letters on the bars within the same storage time denote significant difference (*p* < .05)

The texture of Chinese shrimp depends on the degradation of myofibrillar proteins during storage, which is caused by the microbial decomposition and autolysis after the death of the shrimp. Hardness can be used as a reliable measure for reflecting changes in the shrimp muscle tissue. As shown in Figure 6, firmness in the four groups slightly increased in the first two days, followed by a gradual decrease in hardness, and the decrease was significant in all the four groups. The increase in hardness during the early stages is caused by stiffness of the tissues after shrimp death, while the decrease is caused by autolysis and tissue degradation. Samples in the control group showed the fastest decrease in hardness, followed by samples in the CH group, the CH + ε‐PL + CA group showed the slowest reduction in hardness, which indicated that the complex coating can delay the degradation of myofibrillar proteins caused by intrinsic biological factors, such as enzymes and microbes. Results revealed significant differences in hardness were observed among the four groups from the sixth day (*p* < .05).

#### 3.3.2 Chewiness

**Figure 7:**
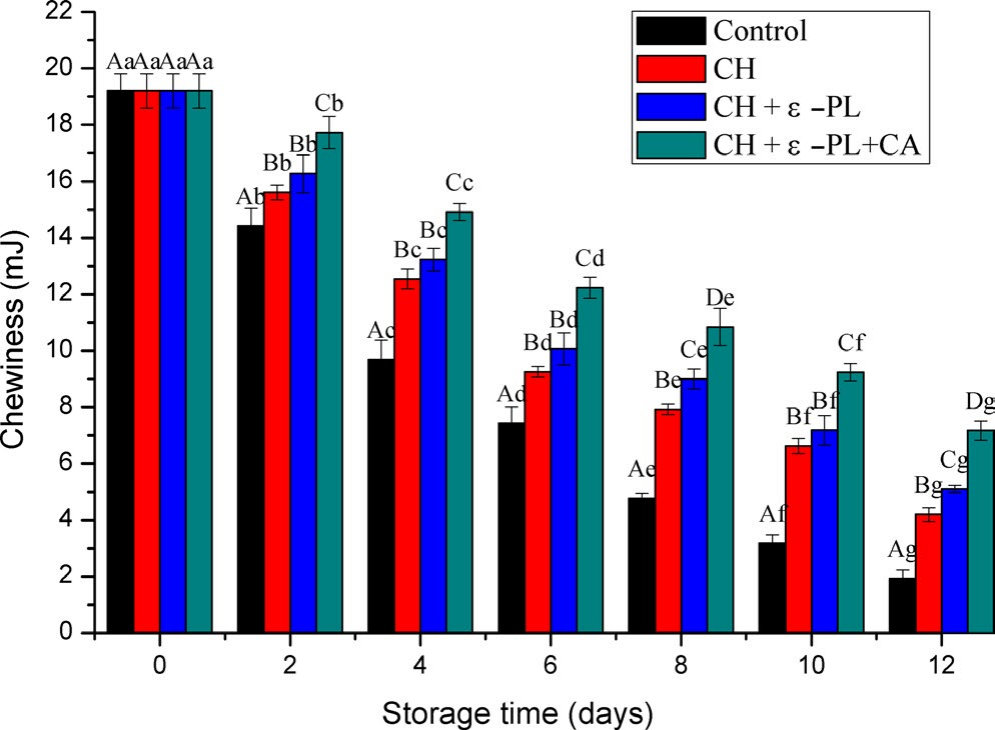
Changes in chewiness scores of Chinese shrimp during refrigerated storage. Different lowercase letters on the bars within the same sample denote significant difference (*p* < .05). Different uppercase letters on the bars within the same storage time denote significant difference (*p* < .05)

Chewiness refers to the energy required to chew semi‐solid samples into a steady state when swallowed. Changes in chewiness of Chinese shrimp during refrigerated storage are shown in Figure 7. A significant decreasing trend in chewiness had been presented for both the treatment and control groups with prolonged storage; however, the control group showed more rapid decrease in chewiness. The molecular structure of collagen changes postmortem, and proteins are gradually degraded and the myofibril structure becomes more loose, leading to softening of the muscles and reduction in chewiness. The three treatments effectively delayed the softening of shrimp muscles. The chewiness of the treated shrimp samples was higher than those of control ones. Variance analysis showed a significant difference in chewiness between CH + ε‐PL + CA group and CH + ε‐PL or CH group (*p* < .05).

#### 3.3.3 Elasticity

**Figure 8:**
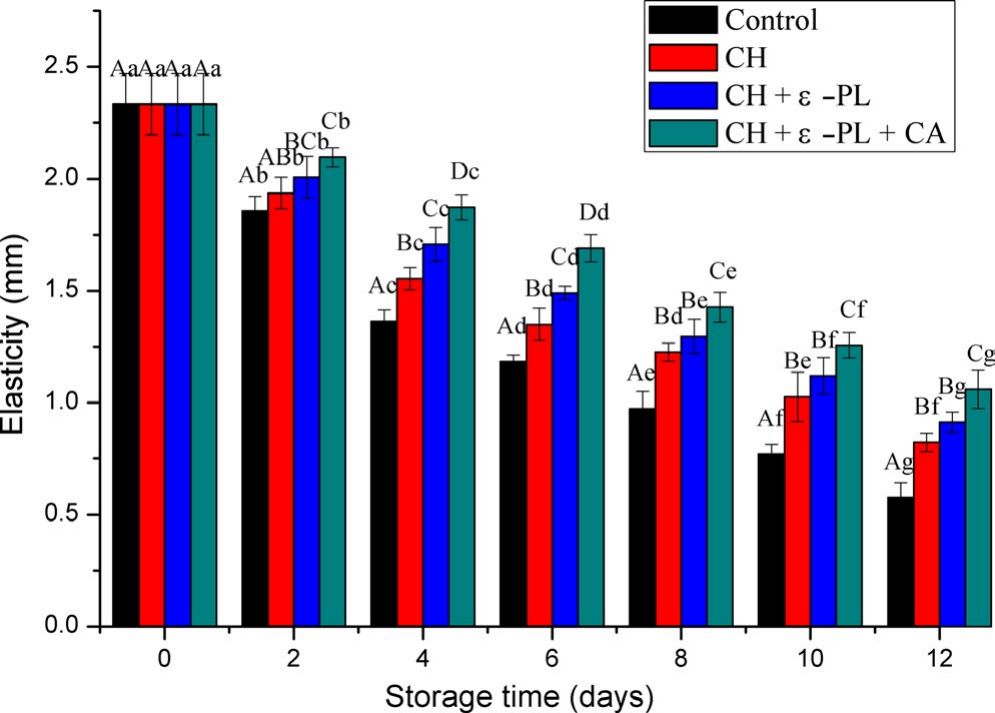
Changes in elasticity scores of Chinese shrimp during refrigerated storage. Different lowercase letters on the bars within the same sample denote significant difference (*p* < .05). Different uppercase letters on the bars within the same storage time denote significant difference (*p* < .05)

As shown in Figure 8, the elasticity decreased with prolonged storage for all samples. Samples in the control group showed the greatest reduction in elasticity, followed by samples in the CH group. On the other hand, samples in the CH + ε‐PL + CA group showed the smallest reductions in elasticity. Results of variance analysis showed that there is a significant difference between the treated groups and untreated control group in terms of elasticity (*p* < .05) from the fourth day. However, on the second, eighth, tenth, and twelfth days, there was no significant difference between the CH group and the CH + ε‐PL group.

Apparently, texture property of Chinese shrimp was improved by coating treatment, while the CH + ε‐PL + CA showed better protective effect than CH or CH + ε‐PL. This is because the stability and rheology of the protective coating solutions were improved by adding carrageenan in contrast to chitosan or chitosan and ε‐polylysine. Hence, the composite coating of the three substances was more effective for delaying the degradation of myofibrillar proteins caused by intrinsic biological factors, such as enzymes and microbes, than the CH or CH + ε‐PL treatments. Similar results showing that the texture deterioration of grass carp fillets was retarded by chitosan‐based coating have also been reported by Yu et al. (2018).

### 3.4 Sensory analyses

**Figure 9:**
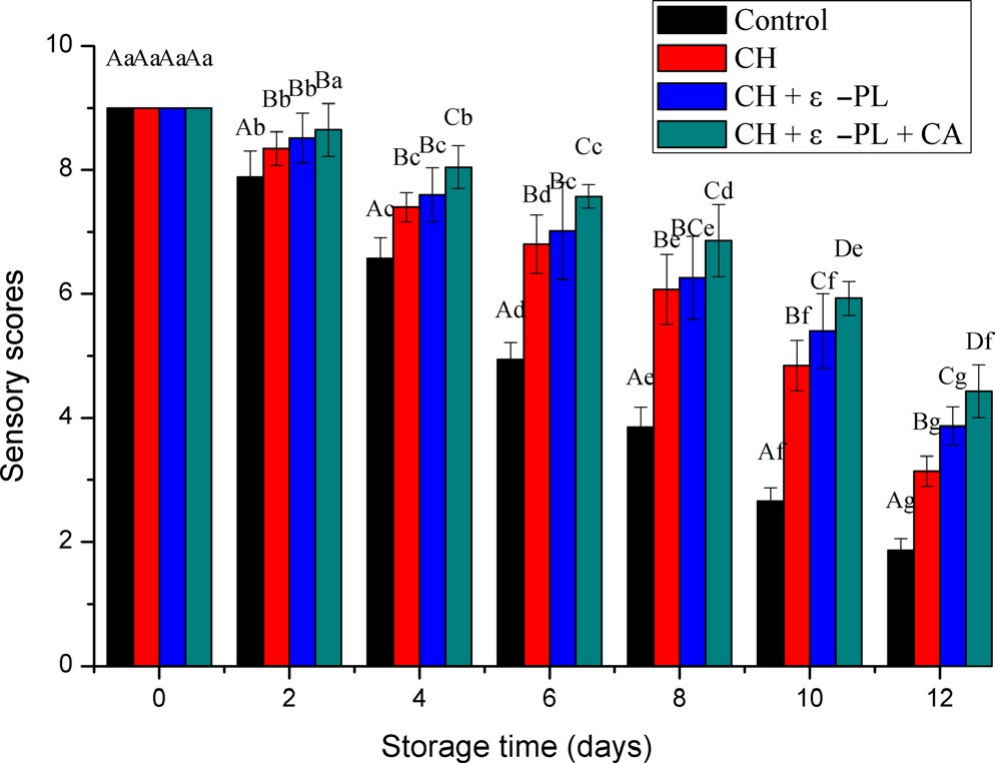
Changes in sensory scores of Chinese shrimp during refrigerated storage. Different lowercase letters on the bars within the same sample denote significant difference (*p* < .05). Different uppercase letters on the bars within the same storage time denote significant difference (*p* < .05)

The sensory profile can indicate the quality of shrimp during storage. According to Yao, Chang, and Wu (2015), shrimp are considered acceptable until the sensory score reached 5.0. As shown in Figure 9, the sensory scores of Chinese shrimp in different treatment groups decreased with prolonged storage, and the decrease was significant in the control, CH, and CH + ε‐PL groups, and in particular, samples in the control group showed the most rapid reduction in sensory scores. Similarly, Hui 2016 found that large yellow croaker treated with chitosan combined with nisin presented better sensory profile compared to the control. The control group was considered inedible at 6th day when the sensory score dropped to 4.94 ± 0.28. By contrast, the sensory scores of the CH and CH + ε‐PL groups were 4.84 ± 0.41 and 5.4 ± 0.6 on day 10, respectively. The above results could be attributed to the protective coating on the surface of shrimp by the chitosan or chitosan and ε‐polylysine, which effectively inhibited microbial proliferation and the protein degradation in shrimp and minimized the production of volatile substances. Samples in the CH + ε‐PL + CA group showed the highest sensory scores during storage among all treatment groups and reached unacceptable values on day 12, indicating that the shrimp shelf life was prolonged by 5 days. The improved preservation is attributed to coating performance and bacteriostasis by the addition of carrageenan to the chitosan and ε‐polylysine. We observed significant differences (*p* < .05) between treated and control groups, but no significant differences (*p* > .05) between CH and CH + ε‐PL groups in the first eight days. The conclusions were consistent with the aforementioned microbiological and chemical quality analyses.

## 4 CONCLUSIONS

The current findings indicated the three treatments with 1.8% CH, 1.8% CH, and 0.13% ε‐PL, or 1.8% CH, 0.13% ε‐PL, and 0.19% CA could maintain the quality of refrigerated Chinese shrimp samples relative to the control treatment. Compared to treatment with CH or CH + ε‐PL, treatment with CH + ε‐PL + CA presented optimal effects in inhibiting the growth of microorganism, decomposition of protein, and oxidation of lipid and in maintaining acceptable characteristics of texture and sensory, and the shelf lives of Chinese shrimp samples were extended by 4–6 days. Therefore, chitosan combined with ε‐polylysine and carrageenan exhibits broad potential as preservation coatings to maintain the quality of Chinese shrimp.

## CONFLICT OF INTEREST

The authors declare that they do not have any conflict of interest.

## ETHICAL STATEMENTS

## Ethical Review

This study does not involve any human or animal testing.

## Informed Consent

Written informed consent was obtained from all study participants.

## References

[fsn31275-bib-0001] Aider, M. (2010). Chitosan application for active bio‐based films production and potential in the food industry: Review. LWT‐Food Science and Technology, 43, 837–842. 10.1016/j.lwt.2010.01.021

[fsn31275-bib-0002] Alasalvar, C. , Taylor, K. D. A. , Öksüz, A. , Garthwaite, T. , Alexis, M. N. , & Grigorakis, K. (2001). Freshness assessment of cultured sea bream (Sparus aurata) by chemical, physical and sensory methods. Food Chemistry, 72, 33–40. 10.1016/S0308-8146(00)00196-5

[fsn31275-bib-0003] Al‐Daqal, M. M. , & Bazaraa, W. A. (1999). Extension of shelf life of whole and peeled shrimp with organic acid salts and bifidobacteria. Journal of Food Protection, 62, 51–56. 10.4315/0362-028X-62.1.51 9921829

[fsn31275-bib-0004] Ando, M. , Nakamura, H. , Harada, R. , & Yamane, A. (2004). Effect of super chilling storage on maintenance of freshness of Kuruma prawn. Food Science and Technology Research, 10, 25–31. 10.3136/fstr.10.25

[fsn31275-bib-0005] Anonymous (2018). Fishery yearbook in China in 2017. Beijing: Chinese Agriculture Press.

[fsn31275-bib-0006] Bico, S. L. S. , Rapaso, M. F. J. , Morais, R. M. S. C. , & Morais, A. M. M. B. (2009). Combined effects of chemical dip and/or carrageenan coating and/or controlled atmosphere on quality of fresh‐cut banana. Food Control, 20(5), 508–514. 10.1016/j.foodcont.2008.07.017

[fsn31275-bib-0007] Carocho, M. , Heleno, S. , Rodrigues, P. , Barreiro, M. F. , Barros, L. , & Ferreira, I. C. F. R. (2019). A novel natural coating for food preservation: Effectiveness on microbial growth and physicochemical parameters. LWT ‐ Food Science and Technology, 104, 76–83. 10.1016/j.lwt.2019.01.031

[fsn31275-bib-0008] Chinese National Standard (2003). Method for analysis of hygienic standard of fish and other aquatic products (GB/T 5009.45‐2003). Chinese National Hygiene Ministry, Beijing.

[fsn31275-bib-0009] Chinese Fisheries Industry Standard (2014). Determination of K value as fishery freshness index‐high performance liquid chromatography (SC/T 3048).

[fsn31275-bib-0010] Chinese National Standard (2015). National standard for food safety fresh and frozen aquatic products (GB 2733‐2015). National Health Commission of the People's Republic of China, Beijing.

[fsn31275-bib-0011] Chinese National Standard (2016). Determination of volatile base nitrogen in foods (GB 5009.228). National Health Commission of the People's Republic of China, Beijing.

[fsn31275-bib-0012] De Abreu, D. A. P. , Losada, P. P. , Maroto, J. , & Cruz, J. M. (2011). Lipid damage during frozen storage of Atlantic halibut (Hippoglossus hippoglossus) in active packaging film containing antioxidants. Food Chemistry, 126, 315–320. 10.1016/j.foodchem.2010.10.048

[fsn31275-bib-0013] Dehghani, S. , Hosseini, S. V. , & Regenstein, J. M. (2018). Edible films and coatings in seafood preservation: A review. Food Chemistry, 240, 505–513. 10.1016/j.foodchem.2017.07.034 28946304

[fsn31275-bib-0014] Ehira, S. (1976). A biochemical study on the freshness of fish. Bull Tokai Regional Fisheries Res Lab, 88, 130–132.

[fsn31275-bib-0015] Fan, W. J. , Chi, Y. L. , & Zhang, S. (2008). The use of a tea polyphenol dip to extend the shelf life of silver carp (Hypophthalmicthys molitrix) during storage in ice. Food Chemistry, 108, 148–153. 10.1016/j.foodchem.2007.10.057

[fsn31275-bib-0016] Genskowsky, E. , Puente, L. A. , Pérez‐Álvarez, J. A. , Fernandez‐Lopez, J. , Muñoz, L. A. , & Viuda‐Martos, M. (2015). Assessment of antibacterial and antioxidant properties of chitosan edible films incorporated with maqui berry (Aristotelia chilensis). LWT‐Food Science and Technology, 64, 1057–1062. 10.1016/j.lwt.2015.07.026

[fsn31275-bib-0017] Günlü, A. , & Koyun, E. (2013). Effects of vacuum packaging and wrapping with chitosan based edible film on the extension of the shelf life of sea bass (Dicentrarchus labrax)fillets in cold storage (4°C). Food and Bioprocess Technology, 6(7), 1713–1719.

[fsn31275-bib-0018] Hui, G. H. , Liu, W. , Feng, H. L. , Li, J. N. , & Gao, Y. Y. (2016). Effects of chitosan combined with nisin treatment on storage quality of large yellow croaker (Pseudosciaena crocea). Food Chemistry, 203, 276–282. 10.1016/j.foodchem.2016.01.122 26948615

[fsn31275-bib-0019] Irma, K. K. , Qurrota, A. , Hanina, M. , & Marsasi, M. R. A. (2019). Encapsulation of Peppermint Oil with Carboxymethyl kappa Carrageenan‐Gelatine‐Chitosan. IOP Conference Series: Materials Science and Engineering, 515, 1–5.

[fsn31275-bib-0020] Jia, S. L. , Liu, Y. M. , Zhuang, S. , Sun, X. H. , Li, Y. , Hong, H. , … Luo, Y. (2019). Effect of ε‐polylysine and ice storage on microbiota composition and quality of Pacific white shrimp (Litopenaeus vannamei) stored at 0 °C. Food Microbiology, 83, 27–35. 31202416 10.1016/j.fm.2019.04.007

[fsn31275-bib-0021] Kirk, R. S. , & Sawyer, R. (1991). Pearson’s composition and analysis of foods (9th edn). London: Longman scientific and technical.

[fsn31275-bib-0022] Kirschnik, P. G. , Viegas, E. M. M. , Valenti, W. C. , & Oliveira, C. A. F. (2006). Shelf‐Life of Tail Meat of the Giant River Prawn, Macrobrachium rosenbergii, Stored on Ice. Journal of Aquatic Food Product Technology, 15(2), 57–71.

[fsn31275-bib-0023] Li, N. A. , Liu, W. R. , Shen, Y. , Mei, J. , & Xie, J. (2019). Coating Effects of ε‐polylysine and Rosmarinic Acid Combined with Chitosan on the Storage Quality of Fresh Half‐Smooth Tongue Sole (Cynoglossus semilaevis Günther) Fillets. Coatings, 9, 1–16. 10.3390/coatings9040273

[fsn31275-bib-0024] Li, T. T. , Hu, W. Z. , Li, J. R. , Zhang, X. G. , Zhu, J. L. , & Li, X. P. (2012). Coating effects of tea polyphenol and rosemary extract combined with chitosan on the storage quality of large yellow croaker (Pseudosciaena crocea). Food Control, 25, 101–106. 10.1016/j.foodcont.2011.10.029

[fsn31275-bib-0025] Li, T. T. , Li, J. R. , Hu, W. Z. , & Li, X. P. (2013). Quality enhancement in refrigerated red drum (Sciaenops ocellatus) fillets using chitosan coatings containing natural preservatives. Food Chemistry, 138, 821–826. 10.1016/j.foodchem.2012.11.092 23411183

[fsn31275-bib-0026] Li, T. T. , Li, J. R. , Hu, W. Z. , Zhang, X. G. , Li, X. P. , & Zhao, J. (2012). Shelf‐life extension of crucian carp (Carassius auratus) using natural preservatives during chilled storage. Food Chemistry, 135, 140–145. 10.1016/j.foodchem.2012.04.115

[fsn31275-bib-0027] Li, T. , Wen, C. , Dong, Y. , Li, D. , Liu, M. , Wang, Z. , … Song, S. (2019). Effect of ε‐polylysine addition on κ‐carrageenan gel properties: Rheology, water mobility, thermal stability and microstructure. Food Hydrocolloids, 95, 212–218. 10.1016/j.foodhyd.2019.04.027

[fsn31275-bib-0028] Li, Y. Q. , Feng, J. L. , Han, Q. , Dai, Z. Y. , Liu, W. , & Mo, H. Z. (2014). Effects of ε‐polylysine on physicochemical characteristics of chilled pork. Food and Bioprocess Technology, 7(9), 2507–2515.

[fsn31275-bib-0029] Li, Y. C. , Yang, Z. Y. , & Li, J. R. (2017). Shelf‐life extension of Pacific white shrimp using algae extracts during refrigerated storage. Journal of the Science of Food and Agriculture, 97(1), 291–298. 10.1002/jsfa.7730 27013186

[fsn31275-bib-0030] Li, Y. N. , Ye, Q. Q. , Hou, W. F. , & Zhang, G. Q. (2018). Development of antibacterial ε‐polylysine /chitosan hybrid films and the effect on citrus. International Journal of Biological Macromolecules, 118, 2051–2056. 10.1016/j.ijbiomac.2018.07.074 30026100

[fsn31275-bib-0031] Lin, L. , Liao, X. , Surendhiran, D. , & Cui, H. Y. (2018). Preparation of ε‐polylysine/chitosan nanofibers for food packaging against Salmonella on chicken. Food Packaging and Shelf Life, 17, 134–141. 10.1016/j.fpsl.2018.06.013

[fsn31275-bib-0032] Lu, S. M. (2009). Effects of bactericides and modified atmosphere packaging on shelf‐life of Chinese shrimp (Fenneropenaeus chinensis). LWT –. Food Science and Technology, 42, 286–291.

[fsn31275-bib-0033] Mehmet, B. , Faruk, B. , & Hami, A. (2009). Preservation and shelf‐life extension of shrimps and clams by high hydrostatic pressure. International Journal of Food Science and Technology, 44, 1495–1502.

[fsn31275-bib-0034] Mu, H. L. , Chen, H. J. , Fang, X. J. , Mao, J. L. , & Gao, H. Y. (2012). Effect of cinnamaldehyde on melanosis and spoilage of Pacific white shrimp (Litopenaeus vannamei) during storage. Journal of the Science of Food and Agriculture, 92, 2177–2182. 10.1002/jsfa.5605 22290525

[fsn31275-bib-0035] Ngo, D.‐H. , Vo, T.‐S. , Ngo, D.‐N. , Kang, K.‐H. , Je, J.‐Y. , Pham, H.‐D. , … Kim, S.‐K. (2015). Biological effects of chitosan and its derivatives. Food Hydrocolloids, 51, 200–216. 10.1016/j.foodhyd.2015.05.023

[fsn31275-bib-0036] Ocaño‐Higuera, V. M. , Maeda‐Martínez, A. N. , Marquez‐Ríos, E. , Canizales‐Rodríguez, D. F. , Castillo‐Yáñez, F. J. , Ruíz‐Bustos, E. , … Plascencia‐Jatomea, M. (2011). Freshness assessment of ray fish stored in ice by biochemical, chemical and physical methods. Food Chemistry, 125, 49–54. 10.1016/j.foodchem.2010.08.034

[fsn31275-bib-0037] Ojagh, S. M. , Rezaei, M. , Razavi, S. H. , & Hosseini, S. M. H. (2010). Effect of chitosan coatings enriched with cinnamon oil on the quality of refrigerated rainbow trout. Food Chemistry, 120, 193–198. 10.1016/j.foodchem.2009.10.006

[fsn31275-bib-0038] Pardio, V. T. , Waliszewski, K. N. , & Zuñiga, P. (2011). Biochemical, microbiological and sensory changes in shrimp (Panaeus aztecus) dipped indifferent solutions using face‐centred central composite design. International Journal of Food Science and Technology, 46, 305–314. 10.1111/j.1365-2621.2010.02474.x

[fsn31275-bib-0039] Plotto, A. , Narciso, J. , Baldwin, E. A. , & Rattanapanone, N. (2006). Edible coating and other surface treatments to maintain color of litchi fruit in storage. Proceedings of the Florida State Horticultural Society. 119, 323–331.

[fsn31275-bib-0040] Qiu, X. J. , Chen, S. J. , Liu, G. M. , & Yang, Q. M. (2014). Quality enhancement in the Japanese sea bass (Lateolabrax japonicas) fillets stored at 4℃ by chitosan coating incorporated with citric acid or licorice extract. Food Chemistry, 162, 156–160. 24874371 10.1016/j.foodchem.2014.04.037

[fsn31275-bib-0041] Roy, S. , & Rhim, J. W. (2019). Preparation of carrageenan‐based functional nanocomposite films incorporated with melanin nanoparticles. Colloids and Surfaces B: Biointerfaces., 176, 317–324. 10.1016/j.colsurfb.2019.01.023 30641303

[fsn31275-bib-0042] Saito, T. , Arai, K. , & Matsuyoshi, M. (1959). A new method for estimating the freshness of fish. Bulletin of the Japanese Society of Scientific Fisheries, 24, 749–750. 10.2331/suisan.24.749

[fsn31275-bib-0043] Sallam, K. I. (2007). Chemical, sensory and shelf life evaluation of sliced salmon treated with salts of organic acids. Food Chemistry, 101, 592–600. 10.1016/j.foodchem.2006.02.019 17245440 PMC1780258

[fsn31275-bib-0044] Sigurgisladottir, S. , Hafsteinsson, H. , Jonsson, A. , Lie, Ø. , Nortvedt, R. , Thomassen, M. , & Torrissen, O. (1999). Textural properties of raw salmon fillets as related to sampling method. Journal of Food Science, 64, 99–104. 10.1111/j.1365-2621.1999.tb09869.x

[fsn31275-bib-0045] Soares, N. M. , Fernandes, T. A. , & Vicente, A. A. (2016). Effect of variables on the thickness of an edible coating applied on frozen fish–Establishment of the concept of safe dipping time. Journal of Food Engineering, 171, 111–118. 10.1016/j.jfoodeng.2015.10.016

[fsn31275-bib-0046] Song, Y. L. , Liu, L. , Shen, H. X. , You, J. , & Luo, Y. K. (2011). Effect of sodium alginate‐based edible coating containing different anti‐oxidants on quality and shelf life of refrigerated bream (Megalobrama amblycephala). Food Control, 22, 608–615. 10.1016/j.foodcont.2010.10.012

[fsn31275-bib-0047] Song, Z. , Li, F. , Guan, H. , Xu, Y. , Fu, Q. , & Li, D. (2017). Combination of nisin and ε‐polylysine with chitosan coating inhibits the white blush of fresh‐cut carrots. Food Control, 74, 34–44. 10.1016/j.foodcont.2016.11.026

[fsn31275-bib-0048] Tavassoli‐Kafrani, E. , Shekarchizadeh, H. , & Masoudpour‐Behabadi, M. (2016). Development of edible films and coatings from alginates and carrageenans. Carbohydrate Polymers, 137, 360–374. 10.1016/j.carbpol.2015.10.074 26686140

[fsn31275-bib-0049] Wu, C. H. , Sun, J. S. , Lu, Y. Z. , Wu, T. T. , Pang, J. , & Hu, Y. Q. (2019). In situ self‐assembly chitosan/ε‐polylysine bionanocomposite film with enhanced antimicrobial properties for food packaging. International Journal of Biological Macromolecules, 132, 385–392. 10.1016/j.ijbiomac.2019.03.133 30904525

[fsn31275-bib-0050] Xu, G. C. , Tang, X. , Tang, S. H. , You, H. B. , Shi, H. W. , & Gu, R. B. (2014). Combined effect of electrolyzed oxidizing water and chitosan on the microbiological, physicochemical, and sensory attributes of American shad (Alosa sapidissima)during refrigerated storage. Food Control, 46, 397–402. 10.1016/j.foodcont.2014.06.010

[fsn31275-bib-0051] Yao, X. C. , Chang, C. F. , & Wu, S. J. (2015). Effect of peach gum polysaccharides on quality changes of white shrimp. International Journal of Biological Macromolecules, 72, 1076–1080. 10.1016/j.ijbiomac.2014.10.024 25450827

[fsn31275-bib-0052] Yoshida, T. , & Nagasawa, T. (2003). ε‐Poly‐l‐lysine: Microbial production, biodegradation and application potential. Applied Microbiology and Biotechnology, 62(1), 21–26. 10.1007/s00253-003-1312-9 12728342

[fsn31275-bib-0053] Yu, D. , Xu, Y. , Regenstein, J. M. , Xia, W. , Yang, F. , Jiang, Q. , & Wang, B. (2018). The effects of edible chitosan‐based coatings on flavor quality of raw grass carp (Ctenopharyngodon idellus) fillets during refrigerated storage. Food Chemistry, 242, 412–420. 10.1016/j.foodchem.2017.09.037 29037708

[fsn31275-bib-0054] Yuan, G. F. , Lv, H. , Tang, W. Y. , Zhang, X. J. , & Sun, H. Y. (2016). Effect of chitosan coating combined with pomegranate peel extract on the quality of Pacific white shrimp during iced storage. Food Control, 59, 818–823. 10.1016/j.foodcont.2015.07.011

[fsn31275-bib-0055] Zhang, B. , Ma, L. K. , Deng, S. G. , Xie, C. , & Qiu, X. H. (2015). Shelf‐life of pacific white shrimp (Litopenaeus vannamei) as affected by weakly acid ice electrolyzed water ice‐glazing and modified atmosphere packaging. Food Control, 51, 114–121. 10.1016/j.foodcont.2014.11.016

